# Supplementary material for: Better accuracy of robotic-assisted total knee arthroplasty compared to conventional technique in patients with failed high tibial osteotomy
Source: PLoS One. 2024 Nov 11;19(11):e0313391. doi: 10.1371/journal.pone.0313391 (PMC11554210; doi:10.1371/journal.pone.0313391)
Supplement: Attachment — Submitted filename: Response to reviewers 20240927.docx [file pone.0313391.s001.docx]

**Response to reviewers**

**Reviewer #1:** 

Dear Authors, I had the opportunity to read your article, which has a clearly defined objective of comparing robot-assisted TKA with conventional TKA in patients with failed HTO. The methodological structure of the study is well articulated, with details on patient selection, inclusion criteria, and procedures performed. The results are clearly presented, with statistically significant differences well highlighted. The detailed description of the surgical procedures for both techniques (robotic and conventional) is useful for understanding the operational differences.

This implies that the physician must necessarily provide appropriate information to the patient about the potential risks, benefits, and specific advantages of one procedure versus the other. In this context, I would suggest an important work that I recommend citing in your references (<https://doi.org/10.1016/j.jflm.2024.102674>), which clearly states that in conducting a thorough informed consent process, physicians have the professional duty to present and initiate a discussion on the risks, benefits, and possible alternatives to a given procedure. Illustrating and discussing possible alternatives is a fundamental element of the disclosure process; patients may not be able to assess risks in abstract terms and would therefore need to rely on a comparative framework to make a truly informed decision. Addressing these discussion elements in detail would certainly contribute to making the article more comprehensive, which is advisable, given that the article, while presenting quality and strengths, needs appropriate elaborations.
**Response: We have added “During this study, we provided sufficient and appropriate information about the respective potential risks, benefits, and specific advantages of robotic and conventional surgeries [15].” in the Materials and Methods.**

**Response: We have added “15. Bolcato V, Franzetti C, Fassina G, Basile G, Martinez RM, Tronconi LP. Comparative study on informed consent regulation in health care among Italy, France, United Kingdom, Nordic Countries, Germany, and Spain. J Forensic Leg Med. 2024;103:102674. doi:10.1016/j.jflm.2024.102674” in the References.**

The inclusion of details such as the measurement of the mechanical axis, alignment, and ligament balancing provides an in-depth view of the methods used. The results showing greater accuracy in mechanical axis and component positioning in the robotic group are well supported by statistical data. The discussion on the importance of alignment and ligament balancing for implant longevity and patient satisfaction is well-argued. In light of these elements—very representative and entirely agreeable—there are some areas that need appropriate elaborations.

In this regard, although the study used a specific robotic system, no detailed information is provided on which system was used. It would be useful to know the type of robotic system employed to better understand the results and their generalizability.
**Response: We have added “The robotic-assisted TKA system (Mako) was introduced to our hospital in 2020. Mako (Stryker, Kalamazoo, MI, USA) is a leading semi-active robotic system and is currently the most commonly used surgical robot for joint operations worldwide [11]. Mako uses preoperative lower extremity computed tomography (CT) images to preplan the size and position of the implant. During surgery, actual knee bone information and preoperative CT data are used to determine the implant size, position, and extent of bone resection. The bone cutting is performed with a cutting saw mounted on the robotic arm. This robotic arm provides haptic feedback and stops the saw when it starts to exceed a preset range while cutting the bone, preventing damage to soft tissue.” in the Materials and Methods.**

**Response: We have added “11. Ma N, Sun P, Xin P, Zhong S, Xie J, Xiao L. Comparison of the efficacy and safety of MAKO robot-assisted total knee arthroplasty versus conventional manual total knee arthroplasty in uncomplicated unilateral total knee arthroplasty a single-centre retrospective analysis. Int Orthop. 2024;48(9):2351-2358. doi:10.1007/s00264-024-06234-0” in the References.**

The lack of clinical follow-up data is a significant limitation. Additional information on long-term clinical outcomes could strengthen the conclusions.
**Response: We have added “The postoperative KSSs in the robotic and conventional groups were not statistically different at a mean follow-up of 2 years (Table 2). The mean KS knee scores in the robotic and conventional groups improved from 36.2 and 35.4 points preoperatively to 79.5 and 75.0 points at the final follow-up, respectively (*p* = 0.121). Mean preoperative function scores in the robotic and conventional groups improved from 38.7 and 38.2 points to 79.8 and 75.8 points at the final follow-up, respectively (*p* = 0.197).” in the Results.**

**Response: We have added “Table 2” in the Results.**

The fact that the study is single-center and retrospective is another limitation. Multicenter and prospective studies could provide more robust and generalizable results.
**Response: We have changed from “This study has several limitations. First, this was a retrospective study and did not include clinical follow-up results. Second, this was a single-center study, and a specific robotic system was used and compared with conventional joint surgery, which may limit the generalizability of the findings to all robotic systems. Finally, plain radiographs were used instead of postoperative CT scans to measure the accuracy of component positioning to reduce the financial burden on patients.” to “This study has several limitations. First, this was a single-center and retrospective study. Multicenter and prospective studies are needed to provide more robust and generalizable results. Second, a specific robotic system was used and compared with conventional joint surgery, which may limit the generalizability of the findings to all robotic systems. Third, plain radiographs were used instead of postoperative CT scans to measure the accuracy of component positioning and reduce the financial burden on patients. Further studies using postoperative CT scans are necessary to confirm and generalize these results because CT scans can provide more precise and detailed measurements.” in the Limitation part.**

The use of simple radiographs instead of postoperative CT scans to measure the accuracy of component positioning is a methodological choice that could be discussed more thoroughly. CT scans provide more precise and detailed measurements, although they may increase costs for patients.
**Response: We have changed from “This study has several limitations. First, this was a retrospective study and did not include clinical follow-up results. Second, this was a single-center study, and a specific robotic system was used and compared with conventional joint surgery, which may limit the generalizability of the findings to all robotic systems. Finally, plain radiographs were used instead of postoperative CT scans to measure the accuracy of component positioning to reduce the financial burden on patients.” to “This study has several limitations. First, this was a single-center and retrospective study. Multicenter and prospective studies are needed to provide more robust and generalizable results. Second, a specific robotic system was used and compared with conventional joint surgery, which may limit the generalizability of the findings to all robotic systems. Third, plain radiographs were used instead of postoperative CT scans to measure the accuracy of component positioning and reduce the financial burden on patients. Further studies using postoperative CT scans are necessary to confirm and generalize these results because CT scans can provide more precise and detailed measurements.” in the Limitation part.**

The mention of intraclass correlation coefficients between the measurements of the two observers is positive, but details on how these coefficients were calculated and interpreted are lacking. More details on these statistical aspects could better clarify the validity of the measurements.
**Response: We have changed from “Differences in variables between the two groups were evaluated using the Mann–Whitney *U* test. Data are presented as the mean ± standard deviation. Intraclass correlation coefficients were calculated using SPSS version 20 to determine the correlation between the measurements of the two independent observers. For all analyses, a *p*-value < 0.05 indicated statistical significance.” to “Student’s t-test was used to analyze age, body mass index, and time from HTO to TKA. The chi-squared test was used to analyze sex, diagnosis, and KS knee and function scores. Data are presented as the mean ± standard deviation. Intraclass correlation coefficients (ICCs) were calculated using SPSS version 20 to determine the correlation between the measurements of the two independent observers. A common approach to quantifying the reliability of a measurement process is to calculate the ICC with a confidence interval.** **ICC is a statistical estimate that measures the degree of agreement between at least two quantitative measurement values. It is designed to measure the degree of reliability, consistency, stability, and agreement. For all analyses, a *p*-value of <0.05 indicated statistical significance.” in the Statistical analysis part.**

It is evident that the surgeon's competence and experience in performing the procedures can significantly influence the results. In conclusion, the article provides a useful comparison between robot-assisted TKA and conventional TKA in patients with failed HTO, highlighting the benefits of robotic assistance in terms of precision and alignment.

However, further studies with long-term clinical follow-ups, use of postoperative CT scans, and involvement of multiple centers are necessary to confirm and generalize these results. Elaboration on methodological and statistical details would further strengthen the study.
**Response: We have changed from “In conclusion, this study showed that robotic-assisted TKA can improve the mechanical axis, increase the accuracy of component positioning and polyethylene liner thickness, and reduce LLD compared with those in conventional TKA in patients with failed HTO. Further larger-scale studies with long-term follow-up measures are necessary to determine whether the accuracy of robotic-assisted TKA can translate into better clinical outcomes and patient satisfaction.” to “Conversion TKA after HTO failure requires careful surgical planning and attention due to the various anatomic variations that occur after osteotomy. This study showed that robotic-assisted TKA could improve the mechanical axis, increase the accuracy of component positioning and polyethylene liner thickness, and reduce LLD compared to those in conventional TKA patients with failed HTO. The application of a robotic-assisted system used in conversion TKA is considered a reasonable technical option for managing failed HTO. Further multicenter studies with long-term follow-up measures are necessary to determine whether the accuracy of robotic-assisted TKA can translate into better clinical outcomes and patient satisfaction.” in the Conclusion part.**

**Reviewer #2:** 

First of all, congratulations on the good results of TKR in HTO failure patients using robots.
Let me point out a few things.

1. Please describe exactly what the definition of failure in HTO.
**Response: We have added “Failure of HTO is defined as the need for conversion to total knee arthroplasty (TKA) since HTO is offered as an option to delay the need for knee arthroplasty.” in the Introduction.**

2. It is unclear whether the advantages of robot surgery in the Ligament balance and the advantages of robot surgery in the valgus state of the tibia due to HTO are advantageous in holding the guide over conventional or in the positioning of the tibial component. Describe the advantages of an accurate robot in this regard.
**Response: We have changed from “Performing conversion TKA after failed HTO requires careful surgical planning and attention because of the various anatomical deformities that occur following osteotomy [7,16]. According to Chen et al. [8], the conversion TKA group after HTO had significantly higher reoperation rates and complications than those in the primary TKA group. They explained that the joint line on the tibial side becomes valgus after osteotomy, and bone deficiencies on the tibial side can be confusing. Using conventional TKA procedures to determine femoral component rotation is often misleading and results in tibial misalignment. Parvizi et al. [7] reported lower Knee Society pain scores in the converted TKA group after HTO with a higher incidence of aseptic loosening during a 15-year follow-up period. Postoperative limb misalignment and poor implant positioning are predictors of implant failure following TKA. Accurate implant positioning and mechanical alignment can improve patient function and implant longevity. Therefore, difficulties, such as achieving accurate alignment and managing unintended anatomical changes after HTO, can be solved using robotic-assisted TKA.” to “Performing conversion TKA after failed HTO requires careful surgical planning and attention because of the various anatomical deformities that occur following osteotomy [7,20]. In this study, robotic surgery showed better accuracy than conventional surgery in terms of the implants needed to restore balance, alignment, and stability. In conventional surgery, especially in failed HTO, where the existing anatomical order was disrupted due to overcorrection or undercorrection, there were many difficulties in determining the amount of tibial cutting due to confusion of the femoral alignment guide and tibial valgus. However, in robotic surgery, the uncertainty of mechanical alignment restoration can be minimized based on preoperative CT images, and ligament balancing and accurate implant positioning can be achieved through real knee bone mapping. Additionally, although not statistically different, KS knee and function scores were higher in the robotic group at a mean follow-up of 2 years. Further long-term follow-up is likely required. According to Chen et al. [8], the conversion TKA group after HTO had significantly higher reoperation rates and complications than those in the primary TKA group. They explained that the joint line on the tibial side becomes valgus after osteotomy, and bone deficiencies on the tibial side can be confusing. Using conventional TKA procedures to determine femoral component rotation is often misleading and results in tibial misalignment. Parvizi et al. [7] reported lower KS pain scores in the converted TKA group after HTO with a higher incidence of aseptic loosening during a 15-year follow-up period. Postoperative limb misalignment and poor implant positioning are predictors of implant failure following TKA. Accurate implant positioning and mechanical alignment can improve patient function and implant longevity. Therefore, difficulties, such as achieving accurate alignment and managing unintended anatomical changes after HTO, can be solved using robotic-assisted TKA. The application of robotic-assisted systems for conversion TKA is considered a reasonable technical option for managing failed HTO.” in the Discussion.**

3. This result does not include clinical results but only radiologic results, such as mechanical axis and component slope. The discussion mentions the long-term follow-up results, but this does not appear appropriate. Please correct it.
**Response: We have added “The postoperative KSSs in the robotic and conventional groups were not statistically different at a mean follow-up of 2 years (Table 2). The mean KS knee scores in the robotic and conventional groups improved from 36.2 and 35.4 points preoperatively to 79.5 and 75.0 points at the final follow-up, respectively (*p* = 0.121). Mean preoperative function scores in the robotic and conventional groups improved from 38.7 and 38.2 points to 79.8 and 75.8 points at the final follow-up, respectively (*p* = 0.197).” in the Results.**

**Response: We have added “Table 2” in the Results.**

4. The analysis of the advantages of robotic surgery in the discussion is not clear. Describe exactly and accureately the advantages of robot-assisted surgery over conventional techniques.
**Response: We have changed from “Furthermore, this study emphasized the advantages of robotic-assisted TKA over conventional TKA in patients with failed HTO with altered anatomy. Robotic-assisted surgery allows for preoperative planning of implant size and position, leg alignment, LLD restoration, and liner thickness by storing CT scans in the robotic system before surgery and also has the flexibility of making appropriate adjustments during surgery. Additionally, in cases where retained hardware is present in patients with failed HTO, it must be removed in conventional TKA before surgery can proceed. However, in robotic-assisted TKA, surgery can be performed without removing the hardware, thereby saving time [23].” to “To summarize, in patients with failed HTO with altered anatomy, robotic-assisted surgery has distinct advantages over conventional surgery. First, preoperative CT images determine surgical planning, including the implant size and position, leg alignment, LLD restoration, liner thickness, etc. Second, data are collected during surgery via real knee bone mapping; therefore, it is possible to combine them with preoperative CT images to determine the final implant position, size, and amount of bone cut without confounding the altered anatomy. Finally, there is an issue that is not specifically addressed in this paper. In cases where retained hardware is present in patients with failed HTO, it must be removed in conventional surgery before surgery can proceed. However, robotic-assisted surgery can be performed without removing the hardware, thereby saving time [27].” in the Discussion.**

**Reviewer #3:** 

We would like to thank the authors for attempting to evaluate robotic technique in TKA, especially after conversion due to HTO.

However, the adequacy of statistical analysis based on tele-radiographs, which do not take into account the possibility of rotational imaging (the gold standard - multispiral computed tomography) due to the human factor and leading to conclusions on the scale of a degree, raises great doubts about their correctness.
**Response: We have changed from “This study has several limitations. First, this was a retrospective study and did not include clinical follow-up results. Second, this was a single-center study, and a specific robotic system was used and compared with conventional joint surgery, which may limit the generalizability of the findings to all robotic systems. Finally, plain radiographs were used instead of postoperative CT scans to measure the accuracy of component positioning to reduce the financial burden on patients.” to “This study has several limitations. First, this was a single-center and retrospective study. Multicenter and prospective studies are needed to provide more robust and generalizable results. Second, a specific robotic system was used and compared with conventional joint surgery, which may limit the generalizability of the findings to all robotic systems. Third, plain radiographs were used instead of postoperative CT scans to measure the accuracy of component positioning and reduce the financial burden on patients. Further studies using postoperative CT scans are necessary to confirm and generalize these results because CT scans can provide more precise and detailed measurements.” in the Limitation part.**

At the same time, the simple assessment of the mechanical axis deviation does not give any insight into the clinical-functional results, which were not evaluated using appropriate scales (example KSS, WOMAC, etc.), which casts doubt on the scientific value of the work, while the number of patients in a methodologically correct approach would allow valuable conclusions to be drawn.
**Response: We have changed from “Propensity score matching was performed using age, sex, and BMI to obtain a comparable control group.” to “The sample size was determined using the G-power program (https://www.g-power.com/en/) [19]. For the sample size analysis, we considered an effect size of 0.5, an alpha significance level of 0.05, and a statistical power of 0.8. The appropriate sample size for the robotic group was determined to be 43 people. The sample size of the robotic group in this study was 30, which may lack statistical power. Therefore, we compared the two groups using propensity score matching to increase statistical power. Propensity scores were calculated for age, gender, and BMI, with robotic and conventional groups matched in a 1:2 ratio.” in the Statistical analysis part.**

**Response: We have added “19. Kang H. Sample size determination and power analysis using the G*Power software. J Educ Eval Health Prof. 2021;18:17. doi:10.3352/jeehp.2021.18.17” in the References.**

The "Aim of the study" is missing in the materials and methods of the main manuscript. The "Discussion" block comes after the "Results", but there are no "Conclusions", which is probably an editing error. The English language needs to be double-checked by a native speaker.
**Response: We have changed from “In conclusion, this study showed that robotic-assisted TKA can improve the mechanical axis, increase the accuracy of component positioning and polyethylene liner thickness, and reduce LLD compared with those in conventional TKA in patients with failed HTO. Further larger-scale studies with long-term follow-up measures are necessary to determine whether the accuracy of robotic-assisted TKA can translate into better clinical outcomes and patient satisfaction.” to “Conversion TKA after HTO failure requires careful surgical planning and attention due to the various anatomic variations that occur after osteotomy. This study showed that robotic-assisted TKA could improve the mechanical axis, increase the accuracy of component positioning and polyethylene liner thickness, and reduce LLD compared to those in conventional TKA patients with failed HTO. The application of a robotic-assisted system used in conversion TKA is considered a reasonable technical option for managing failed HTO. Further multicenter studies with long-term follow-up measures are necessary to determine whether the accuracy of robotic-assisted TKA can translate into better clinical outcomes and patient satisfaction.” in the Conclusion part.**

The reference list needs to be supplemented with current sources, 14 out of 23 references are older than 10 years, including publications that have only museum value in 2024...
**Response: We have changed from “1. Matthews LS, Goldstein SA, Malvitz TA, Katz BP, Kaufer H. Proximal tibial osteotomy. Factors that influence the duration of satisfactory function. Clin Orthop Relat Res. 1988;229:193-200.” to “1. Hamahashi K, Mitani G, Takagaki T, Serigano K, Tani Y, Sato M, et al. Total Knee Arthroplasty Is Superior to Open Wedge High Tibial Osteotomy in Terms of Pain Relief for Patients With Osteoarthritis. Arthroplast Today. 2020;7:7-10. doi:10.1016/j.artd.2020.11.010” in the References.**

**Response: We have changed from “2. Moreland JR, Bassett LW, Hanker GJ. Radiographic analysis of the axial alignment of the lower extremity. J Bone Joint Surg Am. 1987;69(5):745-749.” to “2. Hoorntje A, Pronk Y, Brinkman JM, van Geenen RCI, van Heerwaarden RJ. High tibial osteotomy versus unicompartmental knee arthroplasty for Kellgren-Lawrence grade 3-4 knee osteoarthritis in younger patients: comparable improvements in patient-reported outcomes, adjusted for osteoarthritis grade and sex. Knee Surg Sports Traumatol Arthrosc. 2023;31(11):4861-4870. doi:10.1007/s00167-023-07526-5” in the References.**

**Response: We have changed from “3. Gstottner M, Pedross F, Liebensteiner M, Bach C. Long-term outcome after high tibial osteotomy. Arch Orthop Trauma Surg. 2008;128(1):111-115. doi:10.1007/s00402-007-0438-0” to “3. van WulfftenPalthe AFY, Clement ND, Temmerman OPP, Burger BJ. Survivaland functional outcome of high tibial osteotomy for medial knee osteoarthritis: a10-20-year cohort study. Eur J Orthop Surg Traumatol. 2018;28:1381-1389. doi:10.1007/s00590-018-2199-6” in the References.**

**Response: We have changed from “4. Hui C, Salmon LJ, Kok A, Williams HA, Hockers N, van der Tempel WM, et al. Long-term survival of high tibial osteotomy for medial compartment osteoarthritis of the knee. Am J Sports Med. 2011;39(1):64-70. doi:10.1177/0363546510377445” to “4. Sasaki E, Akimoto H, Iio K, Fujita Y, Saruga T, Kakizaki H, et al. Long-term survival rate of closing wedge high tibial osteotomy with high valguscorrection: a 15-year follow-up study. Knee Surg Sports Traumatol Arthrosc. 2021;29:3221-3228. Doi:10.1007/s00167-020-06128-9” in the References.**

**Response: We have changed from “5. Duivenvoorden T, Brouwer RW, Baan A, Bos PK, Reijman M, Bierma-Zeinstra SMA, et al. Comparison of closing-wedge and opening-wedge high tibial osteotomy for medial compartment osteoarthritis of the knee: a randomized controlled trial with a six-year follow-up. J Bone Joint Surg Am. 2014;96(17):1425-1432. doi:10.2106/JBJS.M.00786” to “5. Yoon JR, Ko SN, Jung KY, Lee Y, Park JO, Shin YS. Risk of Revision Following Total Knee Arthroplasty or High Tibial Osteotomy: A Nationwide Propensity-Score-Matched Study. J Bone Joint Surg Am. 2019;101(9):771-778. doi:10.2106/JBJS.18.00980” in the References.**

**Response: We have changed from “6. Howells NR, Salmon L, Waller A, Scanelli J, Pinczewski LA. The outcome at ten years of lateral closing-wedge high tibial osteotomy: determinants of survival and functional outcome. Bone Joint J. 2014;96-B(11):1491-1497. doi:10.1302/0301-620X.96B11.33617” to “6. El-Galaly A, Nielsen PT, Kappel A, Jensen SL. Reduced survival of total knee arthroplasty after previous unicompartmental knee arthroplasty compared with previous high tibial osteotomy: a propensity-score weighted mid-term cohort study based on 2,133 observations from the Danish Knee Arthroplasty Registry. Acta Orthop. 2020;91(2):177-183. doi:10.1080/17453674.2019.1709711” in the References.**

**Response: We have changed from “9. Haslam P, Armstrong M, Geutjens G, Wilton TJ. Total knee arthroplasty after failed high tibial osteotomy long-term follow-up of matched groups. J Arthroplast. 2007;22(2):245-250. doi:10.1016/j.arth.2006.01.031” to “9. Lee SH, Seo HY, Lim JH, Kim MG, Seon JK. Higher survival rate in total knee arthroplasty after high tibial osteotomy than that after unicompartmental knee arthroplasty. Knee Surg Sports Traumatol Arthrosc. 2023;31(3):1132-1142. doi:10.1007/s00167-021-06641-5” in the References.**

**Response: We have changed from “10. Pearse AJ, Hooper GJ, Rothwell AG, Frampton C. Osteotomy and unicompartmental knee arthroplasty converted to total knee arthroplasty:data from the New Zealand joint registry. J Arthroplast. 2012;27(10):1827-1831. doi:10.1016/j.arth.2012.05.031” to “10. Parente A, Legnani C, Bargagliotti M, Marullo M, Romagnoli S. Medial Unicompartmental Knee Arthroplasty After Failed Open-Wedge High Tibial Osteotomy. J Arthroplasty. 2021;36(8):2746-2751. doi:10.1016/j.arth.2021.03.008” in the References.**

**Response: We have changed from “12. Song EK, Seon JK, Park SJ, Jung WB, Park HW, Lee GW. Simultaneous bilateral total knee arthroplasty with robotic and conventional techniques: a prospective, randomized study. Knee Surg Sports Traumatol Arthrosc. 2011;19(7):1069-1076. doi:10.1007/s00167-011-1400-9” to “12. Li C, Zhang Z, Wang G, Rong C, Zhu W, Lu X, et al. Accuracies of bone resection, implant position, and limb alignment in robotic-arm-assisted total knee arthroplasty: a prospective single-centre study. J Orthop Surg Res. 2022;17(1):61. doi:10.1186/s13018-022-02957-1” in the References.**

**Response: We have changed from “13. Song EK, Seon JK, Yim JH, Netravali NA, Bargar WL. Robotic-assisted TKA reduces postoperative alignment outliers and improves gap balance compared to conventional TKA. Clin Orthop Relat Res. 2013;471(1):118-126. doi:10.1007/s11999-012-2407-3” to “13. Mancino F, Cacciola G, Malahias MA, De Filippis R, De Marco D, Di Matteo V, et al. What are the benefits of robotic-assisted total knee arthroplasty over conventional manual total knee arthroplasty? A systematic review of comparative studies. Orthop Rev (Pavia). 2020;12:8657. doi:10.4081/or.2020.8657” in the References.**

**Response: We have changed from “20. Cerciello S, Vasso M, Maffulli N, Neyret P, Corona K, Panni AS. Total knee arthroplasty after high tibial osteotomy. Orthopedics. 2014;37(3):191-198. doi:10.3928/01477447-20140225-08” to “20. Papp M, Zsákai Z, Gömöri A. Comparison of total knee arthroplasty after combined high tibial osteotomy with a matched group of primary total knee arthroplasty. Eklem Hastalik Cerrahisi. 2019;30(2):79-84. doi:10.5606/ehc.2019.66900” in the References.**

**Response: We have changed from “24. Vaidya SV, Patel MR, Panghate AN, Rathod PA. Total knee arthroplasty: limb length discrepancy and functional outcome. Indian J Orthop. 2010;44:300-307. doi:10.4103/0019-5413.65159” to “24. Begum FA, Kayani B, Magan AA, Chang JS, Haddad FS. Current concepts in total knee arthroplasty : mechanical, kinematic, anatomical, and functional alignment. Bone Jt Open. 2021;2(6):397-404. doi:10.1302/2633-1462.26.BJO-2020-0162.R1” in the References.**

**Response: We have changed from “25. Ulrich SD, Bhave A, Marker DR, Seyler TM, Mont MA. Focused rehabilitation treatment of poorly functioning total knee arthroplasties. Clin Orthop Relat Res. 2007;464:138-145.” to “25. Karasavvidis T, Pagan Moldenhauer CA, Lustig S, Vigdorchik JM, Hirschmann MT. Definitions and consequences of current alignment techniques and phenotypes in total knee arthroplasty (TKA) - there is no winner yet. J Exp Orthop. 2023;10(1):120. doi:10.1186/s40634-023-00697-7” in the References.**
